# Rewriting the Narrative: Advancing Justice and Equity in the U.S. Food System

**DOI:** 10.3390/ijerph22040638

**Published:** 2025-04-18

**Authors:** Marie A. Bragg, Nathalie Lissain, Zora G. Hall, Brittany N. Edghill, Omni Cassidy, Roxanne Dupuis, Karen Watson

**Affiliations:** 1 Department of Population Health, NYU Grossman School of Medicine, New York, NY 10016, USA; nathalie.lissain@nyulangone.org (N.L.); zora.hall@nyulangone.org (Z.G.H.); brittany.edghill@nyulangone.org (B.N.E.); omni.cassidy@nyulangone.org (O.C.); roxanne.dupuis@nyulangone.org (R.D.); 2 Food Environment and Policy Research Coalition, NYU Grossman School of Medicine, New York, NY 10016, USA; 3 Marketing Department, NYU Stern School of Business, New York, NY 10012, USA; 4 Center for Health Economics and Policy Innovation, Imperial College London Business School, London SW7 2AZ, UK; kwatson@kinetic-leaders.com

**Keywords:** food justice, food insecurity, diet-related diseases, U.S. food system, commercial marketing, racial justice, food environment

## Abstract

The concept of ‘food justice’ recognizes the systemic injustices embedded in the U.S. food system and the urgent need for transformative policies to ensure equitable access to affordable, nutritious, and culturally relevant food. Limited access to these foods drives food insecurity and increases the prevalence of diet-related diseases in low-income and minority communities. Dominant narratives that individualize hunger and food insecurity often blame the individual and overlook the underlying structural factors that sustain these issues. These narratives have considerable influence. They shape public opinion and can also guide policy decisions. This commentary explores the goals of the food justice movement in the U.S., describes how the food and racial justice movements intersect, and examines the role of commercial marketing and public policy in shaping the food justice discourse. We also reflect on the efforts that should be made to reframe these dominant narratives and facilitate meaningful change in the food environment.

## 1. Introduction

Food justice is a holistic concept that views healthy, culturally appropriate food as a human right [1]. The food justice movement brings to light the inequities that exist across the food system which comprises all the activities involved in feeding a population, including growing, processing, and selling food [2]. Within this system lies the food environment, which refers to the physical, economic, and social contexts that influence people’s access to food [3].

To fully understand how food justice operates within the food system, it is essential to examine the key factors that shape equitable food environments: access and affordability. Access refers to an individual’s ability to obtain adequate and nutritious food, which is often limited in low-income or marginalized communities [4]. When these individuals lack access to sufficient, safe, and nutritious food, they experience food insecurity—a crisis impacting 13.7 million U.S. households [5,6]. Affordability reflects the economic barriers households face in securing consistent, nutritious food for their families [7]. Food insecurity arises when households have trouble accessing affordable food. Food is also deeply tied to culture and identity. A just food environment provides access to affordable, diverse, and culturally appropriate food options that respect the traditions of communities. Beyond access and affordability, food justice encompasses the ethical production of food, prioritizing environmental sustainability and the protection of the rights, well-being, and safety of food system workers (e.g., farm and restaurant workers). While ethical food production is critical, it falls outside the scope of this paper.

To create meaningful systemic change, the food justice movement requires local and national policies aimed at dismantling inequities in the food system [8]. These policies should seek to address the root causes of food insecurity and create more equitable and sustainable food environments that nourish the body and soul. This commentary examines the goals of the food justice movement in the U.S., its alignment with the racial justice movement, and how commercial and public policy narratives shape the food justice discourse.

## 2. Food Injustice: The Burden of Hunger and Health Inequities in the United States

The U.S. food system leaves many in our society malnourished and unhealthy. While every county and congressional district in the U.S. experiences food insecurity, low-income communities and communities of color are disproportionately impacted by hunger and low food access [9,10]. In 2023, 13.5% of U.S. households were food insecure. Of these households, 5.1% experienced very low food security. Black households had the highest rate of food insecurity at 23.3%, followed by Latinx households at 21.9%, and other racial/ethnic groups at 12.0%, compared to 9.9% of White households [11]. Lower food security is associated with higher rates of chronic diseases, such as hypertension, coronary heart disease, stroke, and cancer, among others [12]. Shame and stigma often surround individuals facing food insecurity. This is largely driven by misleading narratives that frame hunger as a personal failing rather than as a result of economic inequality [13]. Many food-insecure families also encounter judgment when accessing vital resources like the Supplemental Nutrition Assistance Program (SNAP) and food pantries, leading to feelings of isolation when seeking the support they need [14]. Stigma associated with poverty and using food assistance can worsen food insecurity by discouraging participation in aid programs and increasing feelings of shame and embarrassment [15]. Research shows that food assistance programs like the Supplemental Nutrition Assistance Program (SNAP), National School Lunch Program (NSLP), and the Special Supplemental Nutrition Program for Women, Infants, and Children (WIC) are underutilized and 40% of U.S. households do not access these resources [15,16]. Parents and caregivers report feeling ashamed to admit they lack food while others feared being reported to child protective services. These findings highlight the importance of positive marketing strategies to improve awareness of food assistance programs and reduce stigma [15,17].

To address these and other challenges related to food insecurity, the food justice movement encourages communities to take ownership of their food systems [18]. Food activists have introduced terms like “food sovereignty” to advance national dialogue around food justice. Food sovereignty is defined as “[the] people’s right to healthy and culturally appropriate food produced through ecologically sound and sustainable methods” p. 1 [1]. It exists within a food system in which those who produce, distribute, and consume food can also control how it is produced and distributed.

Food justice and food sovereignty underscore the complex history of food and land ownership in the U.S., where exploitation and colonialism have left a lasting legacy [1]. Today, systemic racism, capitalism, and patriarchy sustain racial and economic inequity, directly impacting the food system by creating barriers that disproportionately limit marginalized communities’ access to healthy, nutritious food [19]. In this way, food justice is closely tied to racial justice efforts. The inequitable distribution of food insecurity in the U.S. is rooted in a legacy of discriminatory policies, such as land dispossession and redlining, which further promote segregation and unequal access to resources [20]. Redlining policies still impact urban disadvantaged neighborhoods today [21]. One example is supermarket redlining—the inclination of chain supermarkets to shut down existing stores in impoverished neighborhoods or relocate to affluent suburban neighborhoods—which decreases the accessibility and availability of healthy food choices in disinvested communities [22].

Beyond physical access to healthy food, targeted marketing practices also play a critical role in shaping dietary habits, reinforcing the same systemic inequities that limit food access in marginalized communities. Racially-targeted food marketing, or the advertising strategies companies use to appeal directly to these groups, often targets communities with calorie-dense, nutrient-poor foods, which contributes to diet-related health disparities [23]. The article “Multicultural Marketing: The New Normal for Brands and Consumers” highlights the importance of companies adopting a multicultural mindset to embrace the U.S.’s racial demographic shift [24]. Younger and more brand-aware Black and Latinx consumers shape market trends and are viewed as trendsetters [24]. While this can create greater economic opportunities for companies, it also raises ethical concerns. Consumers have called for greater community advocacy to counteract marketing practices that promote poor diet and adverse health outcomes [25].

## 3. Commercial Marketing and Message Framing: Tools to Advance Food Justice Goals

The food industry is incentivized to market unhealthy foods because they are inexpensive to produce, have high profit margins, and their palatable nature appeals strongly to consumers [26]. Food and beverage marketing on social media has a profound impact on consumer health, and children are especially vulnerable to its persuasive effects [27]. As they spend more time online, their exposure to persuasive advertisements increases, which strengthens brand recognition and can influence eating behaviors [28,29]. Many of these ads often resemble posts from friends, which makes it difficult for adolescents to recognize them as advertisements [29]. Companies have significant resources, including access to consumer data, which they can leverage to personalize their marketing strategies [30]. In contrast, public health campaigns often lag behind due to limited budgets. For example, the National Institutes of Health (NIH) issues grants that are typically a few million dollars each [31], while Coca-Cola alone spends about 4 billion USD annually on marketing [32]. Since corporations have more resources to develop and test commercial marketing messages [33,34], academic researchers who aim to advance a new narrative on food justice may benefit from combining their expertise in message framing with best practices from commercial marketing to humanize food justice narratives. In the same way that marketers use techniques to craft persuasive messages that shape consumer behavior, so too can these strategies be used to disrupt false narratives about those impacted by food insecurity, shifting perceptions away from stigmatizing stereotypes and toward empathy and understanding.

The science of message framing has revealed several approaches to crafting persuasive messages [35]. Research indicates that gain-framed appeals, which emphasize the benefits of adhering to a message’s recommendations, are more compelling than loss-framed appeals, which highlight the disadvantages of noncompliance [36]. Additionally, knowledge and emotional framing are two key approaches for enhancing message persuasiveness. While policymakers and scientists rely on knowledge-based appeals, emotional appeals tend to resonate more with audiences. This shift from emphasizing product qualities, as seen in early advertisements (e.g., Coke adds pep!) [37], to using emotional storytelling highlights the power of appealing to emotions to foster loyalty and influence consumer behavior [38]. Emotion-focused appeals are particularly effective on social media platforms, where influencers—everyday people with large followings—have become powerful figures [39]. Research suggests that social media users form parasocial relationships with influencers. These relationships are one-sided friendships where followers feel closely connected to the influencer due to the authentic, personal nature of the content shared [40]. Influencers, who are often perceived as more trustworthy than traditional celebrities, can play a crucial role in advancing public health narratives, particularly on topics like food justice [41]. Trust and emotional connection are key factors in crafting persuasive messages. By using emotion-based storytelling and influencers, carefully framed messages can emphasize the systemic factors driving food insecurity and highlight the resilience of those affected.

## 4. Reframing Food Insecurity Using Narrative Change

Narrative change involves “disrupting dominant narratives that normalize inequity and uphold oppression and advancing new narratives from our communities [to] imagine a different future” (p. 1) [42]. Damaging narratives about food insecurity and diet-related diseases often frame obesity and hunger as moral failings and disregard the structural barriers that limit access to healthy, nutritious food. This perspective places an unjust burden on individuals [43] and contributes to fragmented understandings of the food system, which shape public opinion and influence policy decisions [44]. Building cohesive, compelling, and positive food justice narratives is essential to better understand the complex challenges of hunger, malnutrition, and obesity.

A key approach to achieving narrative change is through storytelling that centers ethical awareness and human connection. In *Food Justice and Narrative Ethics: Reading Stories for Ethical Awareness and Activism*, Beth Dixon underscores how ethical awareness can drive the creation of new food justice narratives. She describes these narratives as “counter-stories” that challenge dominant frameworks and reveal the identities and circumstances of those striving to nourish themselves” (p.1) [45]. These stories not only humanize individuals experiencing food insecurity but also foster empathy, encouraging the public to take meaningful action. Practical examples of this work are initiatives like StoryCorps’ Supplemental Nutrition Assistance Program (SNAP) Matters Project, supported by the Robert Wood Johnson Foundation [46,47]. This project aims to dismantle harmful stereotypes about individuals who rely on food assistance programs by sharing their lived experiences. Efforts to share these stories on social media demonstrate the power of storytelling in shifting public perceptions and advancing food justice as a societal value [48].

Another way researchers, policymakers, and community organizers can contribute to narrative change is by bridging terminology gaps in discussions around food justice, food sovereignty, and nutrition equity using “impact storytelling” [49]. If different groups use different terms to describe similar issues (e.g., “food apartheid” vs. “food justice”), it can create barriers to uniting around a shared vision. When engaging in discussions around these topics and creating media campaigns aimed at challenging stereotypes, impact storytelling can be used as a powerful tool. Framing messages around shared goals and values is key to having them resonate across diverse audiences [50]. Research suggests that this can result in gradual mindset shifts over time [50]. The public can also play a crucial role by refraining from spreading false narratives—especially narratives that stigmatize people who experience food insecurity and supporting local agencies who work on food justice issues.

Narrative change is a cyclical process involving strategic discussions to align goals and values, audience research to explore perspectives and areas where views can be adjusted, and real-world testing to evaluate the impact of new narratives on attitudes and policy support [44]. By adopting this dynamic process, new narratives that challenge existing stereotypes of food-insecure people can be created.

## 5. Discussion

Promoting food justice is a key component in addressing systemic inequities within the food system and advancing equitable access to healthy and culturally appropriate food. By recognizing food as a fundamental human right, the movement emphasizes the need to dismantle structural barriers that disproportionately impact marginalized communities, such as systemic racism and economic inequality. Achieving food justice involves not only improving access and affordability but also ensuring that food environments respect cultural diversity, uphold labor rights, and prioritize sustainability.

Central to achieving this vision is the role of narrative change, which can reframe public understanding of food insecurity as a systemic issue. Crafting more informed and compassionate public discourse on food justice requires strategic storytelling to advance new narratives surrounding food insecurity. This narrative may express that access to safe and healthy food is a human right or that companies should be held accountable for creating a more equitable food landscape. By leveraging ethical awareness, storytelling, marketing strategies, and cross-sector partnerships, we can reimagine food justice and create a more inclusive food landscape for communities harmed by the U.S. food system.

## 6. Conclusions

Future research could use an experimental survey design to compare the effects of different types of narratives that frame issues around food justice, food security, and potential remedies. Message framing and health communication work has historically been funded by National Institutes of Health, the Robert Wood Johnson Foundation, and other philanthropic groups. Continuing to develop and disseminate effective messages about the goals and purpose of the food justice movement is critical for increasing food security across the United States.

## Data Availability

Not applicable.
